# Supplementary material for: Evaluating the Brexit and COVID-19’s influence on the UK economy: A data analysis
Source: PLoS One. 2023 Jun 15;18(6):e0287342. doi: 10.1371/journal.pone.0287342 (PMC10270588; doi:10.1371/journal.pone.0287342)
Supplement: Attachment — Submitted filename: Evaluating the Brexit and COVID.docx [file pone.0287342.s003.docx]

**"Evaluating the Brexit and COVID-19’s influence on the UK Economy: A Data Analysis"**

The study covers very important and relevant topic. Some suggestions are being provided for considerations.

1. Abstract needs to be rewrite in more cohesive and concise manner.
2. There is need to provide more rationale regarding the choices of economic factors studied in the paper. Why some other factors like inflation, investment are not being studied.
3. There could be some comparative discussion needs to be included in terms of either Brexit influenced economy more or COVID-19 ?
4. There is need to provide rationale regarding the selection of data analysis techniques. Does these techniques enrich study findings.
5. Thorough proofreading of the manuscript is required.
