# Supplementary material for: Evaluating the Brexit and COVID-19’s influence on the UK economy: A data analysis
Source: PLoS One. 2023 Jun 15;18(6):e0287342. doi: 10.1371/journal.pone.0287342 (PMC10270588; doi:10.1371/journal.pone.0287342)
Supplement: Attachment — Submitted filename: Editor Feedback Response.docx [file pone.0287342.s004.docx]

**Editor Response**

2.1) Please ensure that your manuscript is thoroughly copyedited. We feel that the reproducibility of the study is uncertain, as language errors remain.

Response 1: The full manuscript has been edited and updated the language error by English University faculties and Post-Doctoral fellows.

2.2) Please provide a complete data collection strategy in your Methods section, where all data collection is described in detail. Please also state whether all data collection methods complied with the terms and conditions of the data sources.

Response 2:

The study has been classified into three essential areas: development, exchange, and individual (person) of the United Kingdom. It examines how the combined catastrophes affected the UK economy and how individuals responded by utilising industry and region-specific data. Numerous data analysis methods and methodologies, such as neural networks and Google Trend Analysis have been utilized for investigating the future economic variables of the United Kingdom in order to measure the effects of both Brexit and COVID.

This research obtained data extraction procedure and the methodology utilised for the analysis and to investigate the economic trajectory and public reaction in the United Kingdom before and after Brexit and COVID-19. For the study of Brexit and COVID-19, the study has collected the required information (data) for the past decade from 2011 until 2021. This study investigates the differences between Pre-Brexit, Brexit, Post-Brexit, and COVID-19. The required data has been collected from reputable web sources the Office for National Statistics (ONS), Google Trends, and Twitter. ONS is the UK Government website that provides open and transparent access to UK economic statistics. The Office for National Statistics (ONS) is dedicated to being open and transparent about the data they hold. It also shows where that data has come from and gives the main uses of that data. Various data visualisation and data analysis technologies are utilized to examine the obtained data that helps to investigate the impact of Brexit and COVID-19 among the industry sector, and its people of the United Kingdom. In addition, to validate the dataset and forecast the effects of the UK economic conditions on unemployment, GDP, earnings, and trade. The extracting data has been analysed and measured using statistical numerical software. For data forecasting validation and predictive analysis, a neural network model has been proposed using Python.

2.3) Please include in your Data availability statement information on how the code used has been shared, as per PLOS ONE code sharing policies.

Response 3:

This work uses numerical coding. Statistical software employs numerical coding, which enables data transformation, interpretation, and observations. Python was used to create a neural network model to perform forecast validation and predictive analysis. In addition, Python has been utilised to implement the Box-Jenkins method. For Twitter sentiment analysis, this study used the Twitter API and the Text-Blob function in Python.
