# Supplementary material for: Evaluating the Brexit and COVID-19’s influence on the UK economy: A data analysis
Source: PLoS One. 2023 Jun 15;18(6):e0287342. doi: 10.1371/journal.pone.0287342 (PMC10270588; doi:10.1371/journal.pone.0287342)
Supplement: Attachment — Submitted filename: Response to Reviewer 13052023.docx [file pone.0287342.s005.docx]

**Review Feedback Response Version 2**

**Date: 13/05/2023**

**Reviewer #1**: I do not see the reviewer comments being addressed (only the editors’ comments were addressed), so I’m returning with the previous comments for the authors to work on.

**Response:**

Authors are greatly appreciated your cordial response. There appear to be some miscommunication between author and Editor. As per Editor advise, there has been removed ‘Reviewer feedback’ as the ‘Editor Feedback’ was the most recent file. The earlier files were eliminated.

Please see the following email (screenshot) I received 12/04/2023 as an evidence artefact. However, I’ll upload reviewer feedback in older version (21/03/2023) again to the PLOS ONE system.

In addition, authors greatly value your thoughtful suggestions for improving this manuscript by providing additional information into the text and citing the source. The authors have decided to include all pertinent information as per your recommendation.

his paper examines Brexit and COVID-19’s influence on the UK economy. The idea is interesting but the paper could be improved as I will explain below.

- **First**, there is a need to carefully proofread the paper. For example, “last” should be “Last” in the opening of the Introduction.

**Response:**

Thanks for your concern regarding the proofreading. The manuscript has been updated and proofread again as per instruction.

- **Second**, an early study about Brexit in 2018 should be acknowledged so that readers understand when it started, which could also be explained theoretically through the lens of de-internationalization:
- Exiting supranational unions and the corresponding impact on tourism: Some insights from a rejoinder to Brexit. Current Issues in Tourism, 21(9), 970-974.

**Response:**

The UK's EU membership was decided through a June 23, 2016, referendum, with 51.9% voting to leave out of 33,551,983 total votes. This outcome has caused uncertainty in the tourism sector, impacting consumers, industry players, and policymakers.

- A general theory of de‐internationalization. Global Business and Organizational Excellence, 42(2), 9-15.

**Response:**

In the de-internationalization perspective, it was often perceived as a failure, discouraging firms from limiting or discontinuing exports to foreign markets. Definitions of de-internationalization were mostly limited, but some comprehensive ones acknowledge its partial or complete nature. However, they often associate it with voluntariness rather than a strategic response to changes in the global business environment.

- De‐internationalization: An organizational institutionalism perspective. Global Business and Organizational Excellence, 42(3), 58-73.

**Response:**

The lack of transparency in formal policies and their impact creates a growing risk for international business. This risk manifests not only through partial de-internationalization, such as decoupling, but also through complete withdrawal and exit from the international market. Compliance with minimal regulatory requirements poses a significant threat, leading firms to strategically opt for de-internationalization as a means to limit their involvement in international operations.

- **Third**, some lessons from the COVID-19 pandemic should be added:
- History, lessons, and ways forward from the COVID-19 pandemic. International Journal of Quality and Innovation, 5(2), 101-108.

**Response:**

The interdependence of economic and public health has become evident during the pandemic. It highlighted that a nation's economic well-being relies on the health of its citizens, while also emphasizing the global significance of people's well-being. The imposition of lockdown measures worldwide demonstrated the need to pause economies to control the spread of SARS-CoV-2. Although economic health metrics like consumer price index (CPI), gross domestic product (GDP), and unemployment rate are well-established, public health measures can be enhanced by considering contemporary indicators such as emotional well-being and mental health. These measures can have significant implications for human capital and productivity in the economy, especially during times of crisis like the pandemic.

- **Fourth**, some insights on how COVID-19 has impacted on different economies should be added and why a UK focus would add to the field:
- The economic impact of a global pandemic on the tourism economy: The case of COVID-19 and Macao’s destination-and gambling-dependent economy. Current Issues in Tourism, 25(8), 1258-1269.

**Response:** Unlike past crises such as aviation tragedies, natural disasters, and supranational union exits (Huang et al., 2020; Lim, 2017, 2018), the COVID-19 pandemic has had an exceptional impact on the tourism and gambling sectors. Unprecedentedly, widespread lockdowns were implemented in cities and countries, an unprecedented measure. While these actions are vital for saving lives and demand global cooperation to contain infectious outbreaks like COVID-19, they also pose a substantial employment risk, particularly in tourism-dependent cities where it constitutes a primary source of income.

- The quarantine economy: The case of COVID-19 and Malaysia. In COVID-19, Business, and Economy in Malaysia (pp. 3-23). New York: Routledge.

**Response:**

The COVID-19 pandemic led to a global quarantine in 2020, affecting economies worldwide, including Malaysia. The quarantine measures aimed at controlling the public health crisis had mixed effects on economies. The agricultural sector faced significant challenges due to movement restrictions, resulting in disruptions in the farm-to-consumer supply chain. Travel limitations had a notable impact on food delivery and all stages of agricultural production. Although panic buying has diminished and logistical issues for fresh produce are being addressed, there are concerns about the country's ability to maintain food self-sufficiency during future emergencies and potential trade disruptions.

**Fifth**, the conditional approach that is taken to review the impact of Brexit and COVID-19 (i.e., before and after) should be clearly indicated and supported:

Conditional recipes for predicting impacts and prescribing solutions for externalities: The case of COVID-19 and tourism. Tourism Recreation Research, 46(2), 314-318.

**Response:**

Externalities are external events or shocks that can influence the internal operations of organizations and industries. The global impact of the COVID-19 pandemic exemplifies such an externality, affecting a wide range of organizations and industries worldwide. Externalities often drive research and strategic discussions among academics, professionals, and policymakers. However, it is important to recognize the limitations of descriptive and association-oriented research in accurately predicting and prescribing causal effects, as they may lack essential factors like boundary conditions. Drawing causal conclusions from non-causal research designs is inappropriate (Dolnicar, Citation2020).

**Sixth**, since trends and sentiment analysis appear to be of interest, I reckon it would be good to also add some bibliometric insights on Brexit versus COVID-19 research in the UK. Some ideas about bibliometric analysis and its contributions could be found in:

- Guidelines for advancing theory and practice through bibliometric research. Journal of Business Research, 148, 101–115.

**Response:**

This manuscript has already presented a comprehensive analysis including an examination of economic factors, a Neural Network model analysis, and an assessment of the unemployment rate across the entire UK, considering regions and gender. The author is encouraged to incorporate this relevant analysis into future work.

**Seventh**, some insights about the challenges of COVID-19 with regard to the business and economy could be found:

- COVID-19, Business, and economy in Malaysia: retrospective and prospective perspectives. New York: Routledge.

**Response:**

The global COVID-19 pandemic has not only caused a public health crisis resulting in loss of life and widespread suffering but has also severely strained economies worldwide. Lockdown measures were implemented across the globe, including in Malaysia, leading to a temporary halt in economic activities and subsequent reorganization. This unprecedented phenomenon is conceptualized in this chapter as the "quarantine economy."

Although the initial pause was brief, governments gradually reopened their economies while implementing new social practices such as remote work, physical distancing, and visitor records. These measures facilitated the adaptation of economic activities to the ongoing pandemic situation, resulting in a reconfiguration of how businesses operate.

- Transformative marketing in the new normal: A novel practice-scholarly integrative review of business-to-business marketing mix challenges, opportunities, and solutions. Journal of Business Research.

**Response:**

In regard to the Business to Business (B2B) marketing, it has increased focus on marketing issues and their implications in the increasingly turbulent B2B market. It sheds light on B2B marketing strategies that are relevant during major crises, employing the marketing mix as an organizing framework and utilizing the COVID-19 pandemic as a natural context for transformative marketing.

Specifically in the transformative marketing emphasizes "competition" and "superior value," which may contradict the goal of delivering "benefits to all stakeholders" due to potential casualties (e.g., dropouts from competition) or costs (e.g., hidden sacrifices or tradeoffs) associated with such focus. However, the lessons learned from collective efforts in combating the challenges of COVID-19 suggest that "collaboration" and "shared prosperity" are crucial for leveraging limited resources and providing agile and valuable responses. These lessons ensure that no one is left behind during times of adversity.

**Eighth**, some ideas on future research could be added to enrich the paper. Some ideas could be found below:

- Ushering a new era of Global Business and Organizational Excellence: Taking a leaf out of recent trends in the new normal. Global Business and Organizational Excellence, 41(5), 5-13.

**Response:**

The digital economy and advancements in technology have made global business an ever-present force. Firms now have the ability to automate processes, target customers, and access larger global markets. The COVID-19 pandemic has further emphasized the importance of global business, enabling firms not only to survive but also to thrive, contributing to the overall functioning of the economy and society.

What distinguishes the current period from past global crises is the remarkable speed at which firms have shifted from survival mode to pursuing growth and success. Industries like cleaning, delivery, and technology services have showcased exceptional adaptability. The unprecedented changes brought about by the pandemic have created a new normal, demanding the exploration of new ideas and a reassessment of existing ones. This is vital for forging a transformative path forward and attaining organizational excellence in the realm of global business.

- What is at stake in a war? A prospective evaluation of the Ukraine and Russia conflict for business and society. Global Business and Organizational Excellence, 41(6), 23-36.

**Response:**

The authors assert that the topic of Russia and Ukraine contradicts the focus of this manuscript. This paper primarily focuses on the economic impacts of COVID-19 and Brexit in the UK. Consequently, the author is not inclined to include the Russia-Ukraine issue in this particular manuscript. However, this topic could be explored in future research endeavors.
